# Clinical and Structural Results Following Arthroscopic and Open Repair of Isolated Subscapularis Tears

**DOI:** 10.3390/jcm13216589

**Published:** 2024-11-01

**Authors:** Christoph Bartl, Janna Dolde, Florian Gebhard, Stefan Eichhorn, Lisa Hainzer, Stephan Pauly

**Affiliations:** 1 Department of Orthopaedic Trauma Surgery, Ulm University, Albert-Einstein-Allee 23, 89081 Ulm, Germany; sekretariat.unfallchirurgie@uniklinik-ulm.de (J.D.); florian.gebhard@uniklinik-ulm.de (F.G.); 2 Center of Orthopaedic and Sports Medicine, Rosa-Bavaresestr. 1, 80639 Munich, Germany; 3 Department of Biomechanics, TÜV Süd, Munich Ridlerstr. 65, 80635 Munich, Germany; stefan.eichhorn@tuvsud.com; 4 Department of Shoulder and Elbow Surgery, Vivantes Auguste-Viktoria-Klinikum, Rubensstr. 125, 12157 Berlin, Germany; schulter.avk@vivantes.de (L.H.); stephan.pauly@vivantes.de (S.P.); 5 Department of Clinical Medicine, Medical School Berlin, Rüdesheimerstr. 50, 14197 Berlin, Germany

**Keywords:** arthroscopic subscapularis repair, open subscapularis repair, subscapularis test, subscapularis muscle atrophy, subscapularis MRI

## Abstract

**Objective:** With advances in techniques, arthroscopic repair of isolated subscapularis tendon tears has become increasingly popular in recent years. The aim of this study was to analyze the clinical and structural results of arthroscopic repair versus the gold standard of open repair. It is a prospective cohort study with a control group; evidence level III. **Methods:** In a prospective study performed at two centers, 18 patients with an isolated subscapularis tear were treated with arthroscopic repair (ARG) and 16 patients with open repair (ORG) using a uniform single-row suture anchor repair technique in both groups. The subscapularis function was assessed using specific clinical tests (belly-press and lift-off tests), strength testing and shoulder function with the use of the Constant–Murley score (CMS). Standardized magnetic resonance imaging (MRI) was used to evaluate the postoperative subscapularis muscle-tendon status. **Results:** At a minimum follow-up of 48 months, the CMS increased from a mean of 54 points preoperatively to a mean of 86 points postoperatively in the ARG (*p* < 0.01) and from 50 points to 85 points postoperatively in the ORG (*p* < 0.01). Specific subscapularis tests (belly-press test and lift-off test) were significantly improved from the preoperative to the postoperative status in both repair groups (*p* < 0.05). Despite a subscapularis tendon healing rate of over 90% on MRI scans in both repair groups, the incomplete correction of specific muscle tests was a frequent postoperative finding. **Conclusions:** Arthroscopic repair of isolated subscapularis tears achieved equivalent clinical and structural results compared to the gold standard of open repair.

## 1. Introduction

Isolated tendon tears of the subscapularis are relatively rare compared to single tendon supraspinatus tears or combined rotator cuff tears and comprise about 4% of all rotator cuff tears [1,2]. Most subscapularis tears are of degenerative origin and part of a massive rotator cuff tear [1,3]. In contrast, isolated subscapularis tears are often of traumatic origin and show a high percentage of concomitant lesions of the long head of the biceps tendon [1,4,5]. 

The subscapularis musculotendinous unit provides a substantial biomechanical role for the shoulder as the single anterior part of the stabilizing rotator cuff. 

It acts as the main internal rotator of the shoulder and is the single anterior stabilizing part of the transversal force couple of the glenohumeral joint [6]. Untreated, chronic subscapularis tears lead to anterior subluxation of the humeral head and joint degeneration; therefore, subscapularis repair is essential to maintain glenohumeral joint biomechanics [7]. 

Open subscapularis repair was set as the gold standard for the treatment of isolated subscapularis tears over thirty years ago and achieved good-to-excellent clinical and structural results in many studies [1,8,9]. Over the last two decades, arthroscopic rotator cuff repair, especially of superior and posterosuperior tendon tears, along with significant improvements in arthroscopic equipment and fixation techniques, has evolved as the new surgical standard [10,11]. Due to anatomic characteristics with a narrow anterior joint space in the subscapularis region, exaggerated by the increasing swelling during the arthroscopic procedure over time, the transition to an all-arthroscopic subscapularis repair approach was delayed, and open repair still remained the gold standard [12]. With the increasing experience of arthroscopic surgeons at specialized arthroscopy centers, arthroscopic subscapularis repair yielded similar clinical and structural results compared to open repair studies, as shown in outcome studies and three systematic reviews, but still, a comparative study is not available [1,13,14,15,16]. 

The purpose of this comparative study was to evaluate the clinical and structural midterm results following arthroscopic repair compared to the gold standard of open repair for isolated subscapularis tears using a comparable tendon fixation technique. We hypothesized that there would be no significant differences in clinical outcomes and structural integrity between the two groups.

## 2. Materials and Methods 

At two institutions, all patients with an isolated subscapularis tendon tear between 2012 and 2020 were included in our prospective non-randomized study. The intervention was performed by two surgeons at the two centers, both familiar with open and arthroscopic repair techniques (CB and SP). The ethics committee approved the study (university of Ulm; reference 192/14) and written informed consent was obtained from all patients. A total of 18 patients with arthroscopic repair (the arthroscopic repair group—ARG) and 16 patients with open repair (the open repair group—ORG) completed the study and were available for the final follow-up evaluation.

Patients were finally included in the study if an isolated full-thickness subscapularis tendon tear was confirmed at diagnostic arthroscopy. We excluded partial articular-sided subscapularis tears and tears with involvement of the supraspinatus and infraspinatus tendon. All patients underwent subsequent arthroscopic or open tendon repair, applying a uniform single-row suture anchor repair technique at both centers. 

Baseline patient characteristics showed a similar distribution in both groups and are listed in Table 1. 

### 2.1. Clinical Evaluation

Patients were evaluated with the simple shoulder test (SST) and the Constant–Murley score (CMS) preoperatively and postoperatively [17]. Shoulder range of motion, including flexion, abduction and external rotation with the arm at the side, was measured with an electronic goniometer, and internal rotation was evaluated with a hand on the back preoperatively and at follow-up. Specific assessment of the subscapularis was performed with the belly-press test and the lift-off test preoperatively and at follow-up [18]. The belly-press test results were graded as “negative” for a normal test with full ability to maintain internal rotation during the test, “positive” if internal rotation could not be maintained, and “intermediate” if internal rotation could be maintained partially. Manual subscapularis strength testing was performed in the belly-press and lift-off positions in both repair groups at baseline and follow-up, using the standard classification of neurologic assessment with a strength scale from 0 (minimal strength) to 5 (maximal strength).

### 2.2. Magnetic Resonance Imaging 

**Figure 1:**
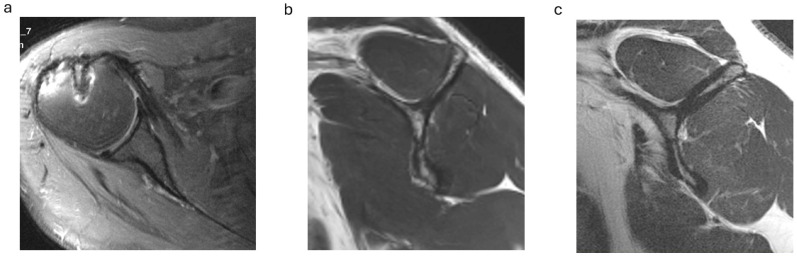
(**a**–**c**). Postoperative subscapularis imaging. (**a**) Intact subscapularis tendon in the axial plane (Sugaya type 1). (**b**) Subscapularis muscle with no atrophy (y-shaped plane). (**c**) Advanced subscapularis atrophy of the upper muscle portion.

**Figure 2:**
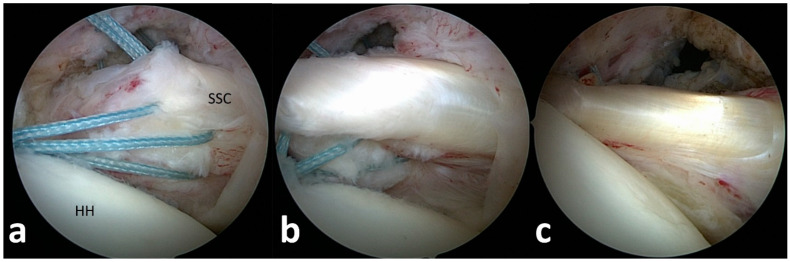
(**a**–**c**). Arthroscopic subscapularis repair technique. (**a**) Arthroscopic tendon identification and release of a type 3 subscapularis tear. (**b**) Arthroscopic tendon mobilization to the lesser tuberosity. (**c**) View from posterior after tendon refixation at the footprint.

Preoperative magnetic resonance imaging (MRI) scans were undertaken to evaluate the tear configuration and subscapularis muscle status with muscle atrophy and muscle fatty infiltration. The fatty infiltration grade (FIG) of the subscapularis muscle was graded using the MRI grading system described by Fuchs et al. based on the adapted Goutallier CT score [19]. Fatty infiltration with FIG 0 and FIG 1 was considered minimal fatty infiltration, representing a normal condition of the study population, FIG 2 moderate and FIG 3 and 4 advanced fatty infiltration of the muscle. 

On postoperative MRI scans, subscapularis tendon repair integrity was graded on axial T2-weighted images using the Sugaya classification, with types 1, 2 and 3 graded as intact tendons (Figure 1a) and types 4 and 5 with tendon discontinuity graded as a retear [20,21]. Subscapularis muscle atrophy was evaluated on the Y-shaped view in the sagittal plane: no atrophy (grade 0), mild atrophy (grade 1), moderate atrophy (grade 2) and advanced atrophy (grade 3) (Figure 1b,c). The MRI scans were graded separately by two investigators (CB and SP) and in consensus in case of discrepancy.

### 2.3. Surgical Procedures

Arthroscopic repair was performed with the patient in the beach chair position. A 30°-angled scope was used through the posterior portal for confirmation of the subscapularis tear and tear grading, as well as the inspection of additional lesions, such as lesions of the long head of the biceps tendon (LHBT) or lesions of the pulley sling. The subscapularis tear size was classified according to the classification of Lafosse et al.: type II tears (complete lesion of the upper third of the tendon), type III tears (complete lesion of the upper two-thirds of the tendon) and type IV tears (complete lesion with retraction but no subluxation of the humeral head). Type I partial tears and type V tears with humeral head subluxation and combined tears of the supraspinatus or the infraspinatus were excluded from the study [5]. 

For tendon mobilization, footprint preparation and anchor insertion, additional anterior and anterolateral portals were established, and the subscapularis tendon edge was identified from the lateral portal (Figure 2). In the case of non-retracted tears of the subscapularis tendon, the connection to the transverse ligament was released for tear confirmation. Retracted tendons were released from scar tissue at the scapular neck, the upper tear margin and anterior scarring to the subcoracoid bursa. The mobilized tendon edge was then pulled with traction sutures to the footprint area at the lesser tuberosity. 

The integrity and stability of the long head of the biceps tendon were inspected with a probe and graded as torn or partially torn, subluxed out of the pulley sling or completely dislocated. 

Tendon refixation was performed using a single-row anchor technique with bioabsorbable suture anchors (Bio-Corkscrew^®^ 5.5 mm; Arthrex, Naples, FL, USA), double-loaded with non-absorbable sutures (Fiberwire^®^; Arthrex). 

Following footprint preparation, one to three anchors, depending on the size of the lesion, were placed from inferior to superior at the lesser tuberosity, and the tendons were fixed with knots after perforation with a suture passer (Figure 2, Video S1). 

A dislocated or partially torn LHBT was either treated with biceps tenodesis or tenotomy according to the functional demands of the patient. 

In the open repair group, a diagnostic arthroscopy was performed to evaluate the subscapularis tear configuration and concomitant lesions. Open tendon repair was performed via a deltopectoral approach with the opening of the rotator interval. The torn and retracted subscapularis tendon was released and refixed with anchors using the same surgical technique as in the arthroscopic group. Following the subscapularis repair, the LHBT was also treated with a tenodesis or tenotomy. 

### 2.4. Rehabilitation

After surgery, the shoulder was immobilized in a shoulder sling in the neutral position for 6 weeks, and external rotation was limited to 0° for 6 weeks. 

After 6 weeks, self-assisted ROM exercises and stepwise external rotation exercises were started. Patients were not permitted to perform heavy work or active exercises with the operated shoulder for 12 weeks. Active strengthening exercises with an elastic band were started after 12 weeks. After 6 months, a gradual return to their premorbid sports activity level and overhead activities of the repaired shoulder were allowed. In both repair groups, the same rehabilitation protocol was applied.

### 2.5. Statistical Analysis 

Statistical analysis was performed using SPSS statistics software (version 26; IBM, Armonk, NY, USA), and the level of significance was set at *p* < 0.05. Data are presented as the mean, standard deviation and range. The paired *t*-test was used for a comparison of pre- and postoperative values. As non-parametric tests, the Wilcoxon test and the Mann–Whitney U test were used to analyze the data. For correlation analysis between samples, the Pearson and Spearman correlation coefficients were applied. 

## 3. Results 

In the ARG, 18 patients completed the final clinical follow-up at an average of 52 months (range: 48–64 months). In the ORG, 16 patients completed the final follow-up at an average of 56 months (range: 48–76 months) after surgery. 

In both repair groups, most patients had a direct or indirect trauma causing the shoulder symptoms (Table 1).

In the ARG, the CMS increased from 54.1 ± 7.2 points preoperatively (range: 43–64) to 86.4 ± 6.2 points postoperatively (range: 69–99) (*p* < 0.001). In the ORG, the CMS improved from 50.4 ± 6.5 points preoperatively (range: 40–65) to 85.1 ± 6.4 points postoperatively (range: 70–98) (*p* < 0.001). There were no significant differences between the ARG and the ORG at the final follow-up (*p* = 0.49) (Table 2). 

The SST increased significantly from 5.8 ± 1.7 points preoperatively to 10.9 ± 1.4 points postoperatively (*p* < 0.001) in the ARG and from 6.1 ± 1.7 points preoperatively to 10.8 ± 1.5 points postoperatively (*p* < 0.001) in the ORG (ARG vs. ORG, *p* = 0.77).

In the postoperative course, the majority of positive or asymmetric preoperative belly-press tests and lift-off tests could be reversed by surgery or at least improved by one category, representing a significant improvement in subscapularis function in both repair groups (*p* < 0.001) (Table 2). 

In the ARG, the preoperative belly-press test was negative in 17%, intermediate in 33% and positive in 50% of cases. Postoperatively, the belly-press test was negative in 61%, intermediate in 22% and positive in 17% of cases. 

In the ORG, the preoperative belly-press test was negative in 13%, intermediate in 31% and positive in 56% of cases. Postoperatively, the belly-press test was negative in 62%, intermediate in 25% and positive in 13% of cases.

The belly-press test reveals a residual partial subscapularis muscle dysfunction of the operated shoulder in both repair groups, resulting in a positive or intermediate postoperative test result in 39% of the ARG and 38% of the ORG (Table 2). Postoperatively, in both repair groups, impaired subscapularis function was not necessarily correlated to a tendon retear, as there was only one tendon retear in each repair group. 

Patients with a positive or asymmetric postoperative belly-press test in the ARG and ORG did not achieve lower CMSs compared to patients with a negative belly-press test (*p* > 0.05 in each group). 

In the ARG, the lift-off test was negative in 56%, intermediate in 27% and positive in 17% in the postoperative course. In the ORG, there were 50% negative, 31% asymmetric and 19% positive lift-off test results. A comparison with preoperative data was not possible for the lift-off test, as the test could not be performed due to limited internal rotation in about 50% of shoulders. 

In the ARG, subscapularis strength in the belly-press position improved from 2.3 (1–5) preoperatively to 4.5 (2–5) postoperatively, and strength in the lift-off position improved from 2.6 (1–5) preoperatively to 4.7 (2–5) postoperatively. 

In the ORG, subscapularis strength in the belly-press position improved from 2.6 (1–5) preoperatively to 4.6 (2–5) postoperatively, and strength in the lift-off position improved from 2.5 (1–5) preoperatively to 4.8 (2–5) postoperatively. 

The measurement of forward flexion and internal rotation showed a significant improvement as a result of the surgical procedure in both groups, whereas external rotation only showed only a mild improvement, mainly due to the fact that some complete type 4 tears showed excessively increased preoperative external rotation (Table 2).

The duration of the surgical procedure of 72 ± 7 min (59–91) in the ARG was not significantly longer compared to the ORG, with a duration of 68 ± 6 min (57–81) (*p* = 0.15).

An average of 2.2 suture anchors (range: 1–3) was used for subscapularis tendon refixation at the lesser tuberosity in the ARG, and in the ORG, an average of 2.4 anchors (range: 1–3) was inserted. In all cases, a complete repair with tendon refixation at the lesser tuberosity could be performed. 

A lesion of the long head of the biceps tendon with subluxation or dislocation out of the bicipital groove was found in 89% (16 of 18) of the ARG. The LHBT was treated with a tenodesis in 13 cases and tenotomy in 3 cases. In the ORG, a lesion of the LHBT with subluxation or dislocation out of the bicipital groove was found in 93% (15 of 16). A biceps tenodesis was performed in 14 cases (Table 3). 

Additional cointerventions were undertaken in the ARG in one case of a labral lesion with an additional arthroscopic capsulolabral repair, one coracoplasty and three subacromial decompressions. In the ORG, one humeral avulsion of glenohumeral ligaments (HAGLs) was refixed at the humeral neck, and one coracoplasty and two subacromial decompressions were performed. 

As complications, there was one tendon retear of the upper portion of the subscapularis tendon in the ARG, and one retear of the upper portion of the subscapularis tendon in the ORG, both confirmed by MRI. 

In one shoulder, 54 months after the subscapularis repair, a new partial bursal-sided tear of the supraspinatus was detected and treated conservatively, in addition to an intact subscapularis repair. One case of postoperative shoulder stiffness in each group in the first year after the procedure was treated conservatively and did not influence the result at the final follow-up. There were no cases of infections, anchor loosening or neurovascular injuries in both repair groups. 

Postoperative MRI grading revealed one (6%) partial subscapularis retear of the cranial subscapularis tendon portion in the ARG and one (6%) partial subscapularis retear of the cranial subscapularis tendon portion in the ORG. 

Subscapularis muscle atrophy grading on the sagittal MRI scans in the ARG group showed no or mild atrophy (grade 0 or 1) in 13 cases (72%) and moderate atrophy (grade 2) in 5 cases. In the ORG, no or mild atrophy was present in 11 cases (69%) and moderate atrophy in 5 cases (Figure 1b,c; Table 3). 

Predominantly subscapularis muscle atrophy in both repair groups was found in the upper subscapularis muscle portion, whereas the inferior part of the subscapularis muscle was not affected significantly in these cases. Higher postoperative atrophy grades of the subscapularis muscle in both groups correlated with a higher rate of positive or intermediate belly-press tests (r = 0.72; *p* < 0.001). On the other hand, higher postoperative subscapularis muscle atrophy grades were not associated with significantly lower postoperative CMSs in both repair groups (*p* > 0.05). 

Preoperative analysis of the subscapularis muscle FIG in the ARG shows an FI grade 0 and grade 1 in 89% and grade 2 in 11% of cases, with a mild progression to an FI grade 0 and grade 1 in 83% and grade 2 in 16% of cases postoperatively. In the ORG, we found a preoperative FI grade 0 and grade 1 in 81% and grade 2 in 19% of cases, with a progression to a postoperative FI grade 0 and grade 1 in 75% and grade 2 in 25% of cases. 

## 4. Discussion

Arthroscopic repair of isolated subscapularis tendon tears achieved equivalent clinical and structural results compared to the established golden standard of open repair. The improvement in overall shoulder function, range of motion and clinical scores was slightly superior in the ARG but not significant. Subscapularis testing and structural MRI results show comparable results in the ARG and the ORG.

Given the rarity of isolated subscapularis tears and that studies with large sample sizes are also scarce, this first comparative study includes quite a large number of patients in both repair groups with a minimum follow-up of 4 years.

In the present study, arthroscopic repair achieved equivalent improvements in the Constant–Murley scores and the SST from the preoperative to the postoperative status compared to the open repair group [1,13,15,22]. Both repair techniques led to a significant regain of specific subscapularis function with a high conversion rate of positive preoperative belly-press/lift-off tests in the postoperative course [1,12,13,15,16]. 

Residual postoperative partial subscapularis muscle dysfunction with incomplete correction of specific subscapularis tests in about 30% of cases was observed in both repair groups [1,13,15]. 

As both repair groups were comparable in terms of tear size, duration from trauma to surgery and the preoperative impairment of subscapularis function (Table 1, Table 2 and Table 3), the type of surgical repair seems to not influence the residual subscapularis dysfunction. In both repair groups, impaired postoperative subscapularis test results correlated with higher atrophy grades of the subscapularis muscle detected on the sagittal MRI scans. 

Both open and arthroscopic repair studies show that large tear sizes, higher degrees of tendon retraction and a longer time interval from injury to surgery, as well as advanced degrees of fatty muscle infiltration of the subscapularis, are negative prognostic outcome factors [23]. 

Independently of the repair technique, postoperative partial subscapularis dysfunction was not associated with significantly reduced CMS, SST or range of motion results or an increased retear rate in both repair groups [12,15,16]. 

Given these findings and the fact that subscapularis function is not represented very specifically in the widely used shoulder scores, evaluation of subscapularis muscle function (belly-press test and lift-off test) and correlation to the subscapularis muscle status (fatty infiltration and muscle atrophy) is mandatory to interpret the clinical and MRI results correctly, as the presence of muscle atrophy or fatty infiltration is not necessarily linked to clinical or structural failure [15,22]. 

In the only available retrospective comparative study, including 22 arthroscopic and 13 open repairs, Nove-Josserand et al. showed comparable CMSs (asc. group: 85 points; open group: 88 points), similar conversion rates of specific subscapularis tests with an improvement in the belly-press results in the majority of cases in both groups and a high healing rate of the subscapularis tendon on MRI scans (asc. group: 86%; open group: 92%) [12]. In their study, progression of the fatty infiltration grade of the subscapularis muscle was found in over 50% of cases in both groups and localized fatty infiltration in the upper subscapularis muscle in over 40% of cases in both groups. This increased fatty muscle infiltration was not associated with lower clinical score results or a higher tendon retear rate on MRI. Incomplete corrections of the belly-press test with a residual subscapularis dysfunction were found in over 30% of cases in both groups but were not associated with lower CMSs or a tendon retear. 

Similar to these findings, we also found in this comparative study that atrophy of the subscapularis muscle was present postoperatively in about 30% of cases in both repair groups. Muscle atrophy was mainly located in the upper muscle part and was associated with an increased rate of positive/intermediate belly-press tests but not with lower CMSs or a tendon retear. The overall fatty infiltration grade of the subscapularis muscle showed slight but not significant progression on the postoperative MRI scans. The cause of this partial muscle atrophy and cases of localized fatty infiltration remains unclear and could be attributed to preoperative muscle disuse atrophy over time in combination with larger tear sizes. Other contributing factors could be an injury during the surgical mobilization procedure or increased tension status in the musculotendinous unit with loss of muscle contractility after the repair. 

Gedikbas et al. and Neviaser et al. reported good clinical outcome results after the arthroscopic and open repair of isolated and combined anterosuperior tears, but the case numbers of isolated tears were too small to perform a comparison analysis between the open and arthroscopic groups [24,25].

Kamijo et al. reported superior outcome results for smaller isolated subscapularis versus larger tears following arthroscopic repair, but partial type 1 and decentered type 5 tears were also included. Larger tears showed inferior results in the ASES (American Shoulder and Elbow Surgeon Score), a lower correction rate in subscapularis tests and a higher retear rate and fatty infiltration grade of the subscapularis detected with MRI [26]. In our study, shoulders with large complete subscapularis tears in both repair groups achieved similar clinical and tendon healing results compared to small- and medium-sized tears. This finding may be attributed to the fact that most large tears were repaired within 6 months before irreversible muscle degeneration and tendon retraction occurred [15,27].

In a retrospective multicenter study, Liu et al. presented ten-year long-term results of arthroscopic (26×) and open (9×) repair of isolated subscapularis tendon tears [28]. The study did not differentiate between the open and arthroscopic repair groups and did not include specific subscapularis tests at the final follow-up. After ten years, the study individuals reached a CMS of 75 points, which is significantly lower than our mid-term results and the average pooled results in the available meta-analysis (arthroscopic repair: avg. 84 points; open repair: avg. 82 points), but shoulder function may have declined over time. They found a comparable rate of subscapularis muscle atrophy/fatty infiltration in about 25% of cases postoperatively, which did not influence the CMS negatively and a still low retear rate of 13% after ten years, confirmed by postoperative MRI.

A hypothesis for the preserved good overall shoulder function and glenohumeral stability shown in many arthroscopic and open repair studies, with a substantial rate of persistent atrophy and fatty infiltration of the subscapularis muscle in 20–50% of cases, is that the intact lower subscapularis muscle can compensate the partial upper muscle atrophy in combination with a remaining tenodesis effect of the upper subscapularis musculotendinous unit [6,14]. 

In this study, we found a low retear rate of 6% in the arthroscopic and open repair groups with a single-row suture anchor repair technique. In the literature, the retear rate following open and arthroscopic repairs of isolated subscapularis tendon tears is reported as 0 to 20% [1,13,15]. These retear rates are far lower than those of isolated supraspinatus tendon tears, which is maybe attributed to the younger patient age and shorter average duration from trauma/symptom onset to surgical repair and, therefore, the better overall quality status of the subscapularis musculotendinous unit [23]. Yoon et al. reported equal clinical and structural results for an arthroscopic single-row anchor repair technique compared to a double-row repair technique for the repair of isolated subscapularis tendon tears [29]. 

In this study, minimally invasive arthroscopic repair with less soft tissue damage achieved slightly better score results and range of motion results compared to open repair but were not significant. Also, the duration of the arthroscopic procedure could be improved in contrast to former comparative studies, like the UKUFF trial that reported a significantly longer operative time for arthroscopic repair [30]. In this study, the duration of the arthroscopic procedure was not significantly longer than that of the open repair procedure. On the other hand, the costs of arthroscopic rotator cuff repair are reported to be higher than those of comparable open repair procedures due to specific arthroscopic instruments, fixation devices and single-use instruments [30]. In this study, we can report a significant improvement in the shoulder outcome scores (CMS and SST) in both groups. The arthroscopic repair group achieved equal improvements looking at the MCID (minimal clinically important difference) and the SCB (substantial clinical benefit) of the clinical scores from the preoperative to the postoperative status [31].

This study has several limitations. First, it was a comparative, prospective study with no randomized patient assignment, and the MRI assessment was not blinded. The statistical power of the study was limited by the relatively small number of subjects. As strengths, we performed a uniform tendon fixation technique in both groups, and the outcome assessment was performed by assessors who were not members of the surgical team. A potential advantage of arthroscopic subscapularis repair is that concomitant intraarticular injuries, like capsulolabral lesions, can be addressed directly, and less soft tissue damage and scarring leads to faster regain of a range of motion and lower rates of shoulder stiffness in the early postoperative course.

## 5. Conclusions

In summary, we found in this first comparative study that arthroscopic repair achieved equivalent clinical mid-term results compared to open repair. Also, tendon healing and the muscle status of the subscapularis, as detected by MRI, showed comparable results following both repair techniques. Arthroscopic subscapularis repair can also be considered a surgical standard for the rare and complex entity of isolated subscapularis tears. 

## Figures and Tables

**Table 1:** Preoperative patient demographics.

	Arthroscopic Repair Group	Open Repair Group	*p* Value
Number of shoulders	18	16	
Mean age, y (range)	51 (28–62)	54 (33–66)	0.52
Gender			
Male/Female	78%/22%	81%/19%	0.81
Duration Symptomatic onset To surgery (months)	5.5 (1–9)	6.8 (0.5–11)	0.16
Traumatic tear (%)	89%	81%	0.87
Dominant side affected (%)	76%	71%	0.61
Tear size Lafosse classification			
Type II	5	4	
Type III	7	6	
Type IV	6	6	

**Table 2:** Pre- and postoperative clinical results. CMS given in points as the mean ± standard deviation (range), and flexion and external rotation given in degrees as the mean ± standard deviation (range). Internal rotation is given as points according to the CMS, and belly-press test given as portion of positive/intermediate test results.

	Arthroscopic Repair Group (n = 18)	Open Repair Group (n = 16)	*p*-Value
Constant–Murley score			
Preoperative	54 ± 7 (43–64)	50 ± 6 (40–65)	0.07
Postoperative	86 ± 6 (69–99)	85 ± 6 (70–98)	0.49
*p*-value	*p* < 0.001	*p* < 0.001	
Flexion			
Preoperative	128° ± 22 (63–151°)	123° ± 23 (58–155°)	0.54
Postoperative	168° ± 14 (121–180°)	164° ± 13 (125–180°)	0.31
*p*-value	<0.001	<0.001	
External rotation			
Preoperative	45° ± 16 (24–90°)	43° ± 18 (21–95°)	0.61
Postoperative	59° ± 9 (31–78°)	55° ± 9 (28–71°)	0.08
*p*-value	<0.001	<0.001	
Internal rotation			
Preoperative	5.6 ± 1.8 (2–8)	5.4 ± 1.7 (1–8)	0.63
Postoperative	7.6 ± 1.5 (4–10)	7.3 ± 1.6 (3–10)	0.59
*p*-value	0.001	0.001	
Belly-press test Positive/Intermediate Test result			
Preoperative	15/18 (83%)	14/16 (88%)	0.85
Postoperative	7/18 (39%)	6/16 (38%)	0.93
*p*-value	*p* < 0.001	*p* < 0.001	

**Table 3:** Surgical procedure and postoperative MRI results.

	Arthroscopic Repair Group (n = 18)	Open Repair Group (n = 16)	*p*-Value
Operative time (min)	72 ± 7 (59–91)	68 ± 6 (58–81)	0.15
LHB treatment (n/%)			
Tenodesis	13/72%	14/88%	
Tenotomy	3/17%	1/6%	
Torn	2/11%	1/6%	
Subscapularis retear rate (n/%), MRI	1/6%	1/6%	
Subscapularis muscle atrophy, MRI			
No or mild atrophy	72%	75%	
Moderate or advanced atrophy	28%	25%	

## Data Availability

All data are contained within the article.

## Supplementary Materials

The following supporting information can be downloaded at: https://www.mdpi.com/article/10.3390/jcm13216589/s1, Video S1: Arthroscopic subscapularis repair.
